# Effects of Eight-Week Game-Based High-Intensity Interval Training Performed on Different Pitch Dimensions on the Level of Physical Capacity and Time-Motion Responses in Youth Soccer Players

**DOI:** 10.5114/jhk/190842

**Published:** 2024-12-19

**Authors:** Zbigniew Jastrzębski, Dorota Wakuluk-Lewandowska, Ersan Arslan, Bulent Kilit, Yusuf Soylu, Łukasz Radzimiński

**Affiliations:** 1 Department of Physiology, Gdansk University of Physical Education and Sport, Gdańsk, Poland.; 2 Faculty of Sport Sciences, Tokat Gaziosmanpasa University, Tokat, Turkey.

**Keywords:** football, small-sided games, physiological responses, external load

## Abstract

This study aimed to assess the effects of two different pitch dimensions on physical fitness responses and time-motion characteristics in young soccer players during the in-season period. Thirty-nine participants were randomly assigned to two playing areas: a 90 m^2^ (PD90, n = 19) and a 180 m^2^ (PD180, n = 20) area per player. During the eight-week soccer-specific high-intensity interval training (HIIT) intervention, both groups performed four bouts of 5-a-side games (SSGs) in two different pitch dimensions of 5 min with 3 min active rest intervals between games. The heart rate (HR), total distance (TD) and distances covered while walking (WLK), jogging (JOG), low-speed running (LSR), high-speed running (HSR), and sprinting (SPR) were recorded during SSGs. Before and after the intervention, the following tests and variables were completed and evaluated: physical work capacity (PWC170), the Wingate anaerobic test, the lactate threshold (LT), 5-m, 20-m, and 30-m sprint tests. Significant between-groups differences were found post-intervention in PWC170 (p = 0.018, ES = 0.15) and the 5-m sprint (p = 0.002, ES = 0.24). Greater playing areas could be more beneficial in developing aerobic fitness, while SSGs on smaller pitch sizes improve the ability of short-distance accelerations.

## Introduction

Soccer is usually categorized as a physically demanding team sport characterized by more than 1000 activities (e.g., walking, jogging, running, sprinting, jumping, accelerations, decelerations etc.) performed during the 90-min match (Stolen et al., 2005). Nevertheless, these demands vary depending on the players’ age. Although the game duration in youth soccer could be shorter (between 60 and 90 min), the level of aerobic capacity is relatively high. Previous studies (Blecharz et al., 2022; Higino et al., 2017; Mandroukas et al., 2021; McMillan et al., 2005; Papla et al., 2022) have demonstrated that maximal oxygen uptake (VO_2max_) in elite young players is close to 60 ml/kg/min or even higher.

Currently available technologies allow for accurate evaluation of physical match performance in youth soccer. Palucci Vieira et al. (2019) in their systematic review showed that running performance during the game was age-related. In general, older players covered longer total distance (TD) and distance in high-intensity zones. Moreover, similar differences were found according to the level of competition. Greater physical match performance was observed in elite young players in comparison with non-elite or recreational players. The TD covered by players at the age of 15–18 years old was within the range from 7100 m to 11500 m what corresponded with relative distance between 99 m/min and 121 m/min (Buchheit et al., 2010; Goto and Saward, 2020; Pereira Da Silva et al., 2007; Pettersen et al., 2019). Furthermore, top speed reported for players at this age was between 26 and 31 km/h (Al Haddad et al., 2015; Mendez-Villanueva et al., 2011). Preparing young players to such high-demanding game requires regular performance of high-intensity efforts.

One of the most commonly used training methods for effective improvement of athletes’ physical performance is high-intensity interval training (HIIT). HIIT is usually defined as either repeated short to long bouts of rather high- but not maximal-intensity exercise interspersed with incomplete recovery periods (Buchheit and Laursen, 2013). Depending on the nature of the intervention, HIIT involves efforts lasting up to 5 min (Gibala, 2021). The effectiveness of short-term interval interventions caused this training to become very popular and widely used in athletes from different sports (Koral et al., 2018; Kostikiadis et al., 2018). Numerous studies have analyzed the effect of different forms of HIIT on soccer players (Arslan et al., 2020; Hill-Haas et al., 2009; Jastrzebski et al., 2014). Over two decades ago, Helgerud et al. (2001) demonstrated that eight weeks of running HIIT improved aerobic fitness and physical match performance in young soccer players. Further comparative research showed that soccer-specific interval training (small-sided games; SSGs) could be even more effective in developing players' VO_2max_, power and technical skills than traditional running efforts (Radziminski et al., 2013). Game-based training exercises involve a large number of soccer-specific movements (such as accelerations, decelerations, and changes of direction). Therefore, due to several physical benefits and greater players’ enjoyment, soccer coaches prefer to apply SSGs instead of traditional interval running exercises (Los Arcos et al., 2015). Nevertheless, practitioners should be aware that during SSGs, standardization of the load is very difficult because of highly variable individual responses of players (Buchheit and Laursen, 2013). Thus, a hybrid approach that includes both, game-based and isolated exercises increases the chance of applying similar training volume and intensity. Modern technologies allow for preparing precise and individual (e.g., based on the match performance or the playing position) training loads for each player. Furthermore, the possibility of real-time monitoring enables the evaluation of physical performance during the training session. If a player does not complete the pre-training assumptions, coaches are able to implement additional isolated compensating efforts.

The intensity of 90–95% HR_max_ during HIIT bouts was previously found to be effective in developing aerobic fitness in young soccer players (Helgerud et al., 2001). Among several variables, the dimensions of the pitch were identified as an useful tool for manipulating the intensity of SSGs (Rampinini et al., 2007). Castillo et al. (2021) reported that a greater area per player (200 m^2^ vs. 100 m^2^) resulted in longer TD and distance covered in high speed zones. Similarly, higher top speed values were reached by players during SSGs performed on larger fields. Another research (Guard et al., 2022) highlighted increased physiological, mechanical and perceptual responses with larger playing areas. Furthermore, Castillo-Rodriguez et al. (2023) demonstrated that areas per player between 150 m^2^ and 250 m^2^ were most suitable to replicate the official match demands in young soccer players. All abovementioned studies refer to acute responses to SSGs performed in various formats. However, the chronic effect of these acute differences is still unknown. Thus, adjusting the pitch size and selecting an appropriate area per player are basic tasks for soccer coaches when planning SSG training sessions.

A typical soccer season consists of three training periods (pre-season, in-season and off-season). Most intermittent training interventions are applied in the pre-season period when training volume is higher and players do not compete in league games. These programs usually last 4–8 weeks and involve two or three experimental training sessions a week (Clemente et al., 2022; Dellal et al., 2012; Ortiz et al., 2024; Radziminski et al., 2013). Furthermore, Moran et al. (2019) suggested in their meta-analysis that each SSGs session should comprise at least 4 sets of 4 min of activity with 3 min of active recovery. Although SSG sessions are commonly used during the in-season period, the number of studies describing such training interventions is very low (Dello Iacono et al., 2021). Such challenges as unequal match-related fatigue or variable structure of the weekly microcycle make long-term experiments difficult to complete. Thus, studies introducing soccer-specific HIIT performed during the competitive season are valuable.

The current study aimed to compare the effects of two eight-week game-based HIIT interventions on physical fitness and time-motion responses during the in-season period in young soccer players. It was hypothesized that SSGs performed on a larger pitch dimension (180 m^2^ per player, (PD_180_)) would develop players’ aerobic fitness more effectively than small relative pitch areas (90 m^2^ per player (PD_90_)). To the best of our knowledge, this is the first study to compare the effectiveness of in-season soccer-specific HIIT in different pitch areas.

## Methods

### Study Design

The present research consisted of two parallel eight-week HIIT interventions during the in-season period (from September to November). After completing the pre-intervention tests, players were assigned to one of the two subgroups. Training loads applied to both groups were identical except for the intervention training sessions performed on Tuesdays (match day −4, three days after the previous league game, Table 1). During this session, after the warm-up (jogging, muscle activation, soccer specific exercises with the ball, dynamic stretching, linear and non-linear accelerations), participants completed 5-a-side games with goalkeepers (4 games of 5-min duration interspersed with 3 min of active recovery). One of the groups (PD_180_) performed the games on the 40 x 45-m field, while the second group (PD_90_) trained on the 28 x 32-m field. The number of ball touches was unlimited and coaches were responsible for proper ball distribution and players’ encouragement during the game. All the games were performed on a synthetic pitch. The study protocol was integrated with the standard training program of the club. A typical weekly microcycle consisted of five training sessions (Monday–Friday) and one league match (Saturday). No training/matches were applied on Sunday. After eight weeks of HIIT, the post-intervention tests were performed. Moreover, the distance covered by players during the first and last SSGs was compared. All the tests were performed within three days. The aerobic and anaerobic assessment took place in the laboratory on day 1, while the lactate threshold (LT) was determined on the synthetic soccer pitch on day 2, and sprint tests were completed on day 3. According to the Declaration of Helsinki, players and their legal guardians were fully informed about the study design and signed a written informed consent form. Moreover, the research protocol was approved by the Ethics Committee at the District Medical Chamber in Gdańsk, Poland (agreement number: KB: 19/16; approval date: 13 September 2016).

**Table 1:** Typical weekly training content distribution during eight weeks of intervention.

Days of the week	Training content	
	Morning session	Afternoon session
Monday	Technical, tactical, endurance (aerobic intensity)	-
Tuesday	Speed, small-sided games (intervention training session)	Individual training session
Wednesday	Coordination, large games	Individual training session
Thursday	Speed, plyometric, tactical	-
Friday	Coordination, speed, tactical	-
Saturday	League game	-
Sunday	-	-

### Participants

Before the study began, the sample size was estimated using G*Power software (G-Power, version 3.1.9.7, University of Dusseldorf, Dusseldorf, Germany). After adding a partial effect size of 0.30, a power of 0.8, a *p*-value of 0.5 (two groups and two measurements), and a correlation of 0.5, a total sample size of 24 was required. Therefore, thirty-nine young male soccer players were randomly divided into two subgroups: PD_90_ (*n* = 19, age: 16.2 ± 0.4 years, body height: 177.1 ± 6.5 cm, body mass: 65.8 ± 11.6 kg) and PD_180_ (*n* = 20, age: 17.2 ± 0.5 years, body height: 176.8 ± 5.4 cm, body mass: 69.4 ± 6.9 kg). After the initial testing phase, players were randomly assigned to one of the two intervention groups by tossing a coin. Then the between-group comparison was performed to exclude possible differences in the baseline level of physical fitness. Neither the researchers nor participants were blinded during the intervention. However, researchers responsible for conducting the physical fitness tests had no knowledge about the group allocation. The average training experience of the participants was 6.2 ± 0.5 years. All the players attended the same sports school and were members of the same club which designated two separated teams for the league competition. To ensure equal training loads, compensation training sessions (including friendly matches or large training games) were performed by players who played less minutes in official matches. The average players’ maximal running speed (V_max_) was 8.30 ± 0.52 m∙s^−1^ and running velocity at the lactate threshold (V/LT) was 3.51 ± 0.24 m∙s^−1^.

### Aerobic Fitness Evaluation

The physical working capacity test (PWC 170) was performed in the laboratory (temperature: 20–21°C, pressure 1015–1020 hPa, humidity of 40–45 %) to evaluate the level of aerobic fitness. The test consisted of 10 min of ergometric work (60 revolutions per minute, RPM) on a stationary bicycle (Monark 894E, Sweden) divided into two stages with an individually determined progressive load (first stage: 1.5 W∙kg^−1^; second stage: 2 W∙kg^−1^). The internal response was recorded at the end of each stage using HR monitors (Polar Electro OY, Kempele, Finland, 2013). The PWC 170 value (expressed in kGm•min^−1^) was considered the aerobic fitness indicator. This indirect test allowed for VO_2max_ estimation based on the following formula: VO_2max_ = 1.7 × PWC170 + 1240 (Jastrzębski et al., 2011). This method was previously identified as a valid predictor of VO_2max_ (Boreham et al., 1990).

### Anaerobic Fitness Assessment

Peak power (W•kg^−1^) was assessed using a 30-s Wingate test on a cycle ergometer. A relative load corresponding to 7.5% of the player’s body mass was applied. Before performing the test, participants completed a 10-min warm-up, including pedaling at a frequency of 60 RPM, with a relative load of 1.2 W∙kg^−1^ and three rapid accelerations between the 7^th^ and the 10^th^ min followed by 5 min of stretching exercises. All players were verbally encouraged by the coach and by the person conducting the test. After 30 s of this supramaximal effort, participants continued cycling with a relative load of 0.5 W∙kg^−1^ to remove the accumulated lactate from the muscles. The highest value of produced power expressed in W∙kg^−1^ was considered as peak power.

### Lactate Threshold Determination

The lactate threshold was determined one week before the first SSG training session using an incremental running test (Radzimiński et al., 2010). To ensure the full recovery, players participated in the test 96 h after the last game. Running speed increased gradually by 0.4 m∙s^−1^ starting from 2.8 m∙s^−1^ to exhaustion. Blood samples were collected from the fingertip to determine the actual lactate concentration. Such variables as running velocity and the HR were used to describe the intensity at the lactate threshold (V/LT and HR/LT, respectively). Moreover, individual HR_max_ values were determined during the test.

### Sprint Tests

Sprint tests were performed on an artificial pitch using photocells (Smartspeed, Fusion Sport, Cooper Plains, Australia). Speed assessment was preceded by a 20-min warm-up involving general exercises, dynamic stretching, activation, and short (5–10 m) and long (20–30 m) distance accelerations. Every player completed two runs at maximal intensity. The starting line was set 0.6 m before the first gate. Players were verbally encouraged to complete the 30-m sprint as fast as possible. Moreover, the split time at 5 m was recorded. V_max_ was determined using global positioning system (GPS) devices during additional 40-m sprints. The rest interval after each 30-m and 40-m sprint was 3 and 4 min, respectively. The final analysis included better (faster) times obtained by players in both trials.

### Time-Motion Analysis

The external load data were collected using a GPS system (minimax 4.0, Catapult Innovations, Melbourne, Australia). This previously validated system was activated 5 min before each training session according to the producer's recommendation. Speed zones were set as follows: walking (WLK, 0–1 m∙s^−1^), jogging (JOG, 1–2 m∙s^−1^), low-speed running (LSR, 2–4 m∙s^−1^ − V/LT), high-speed running (HSR, V/LT – 80% V_max_), and sprinting (SPR > 80% V_max_) (Jastrzębski and Radzimiński, 2015). The GPS devices were compatible with Polar straps and H7 sensors which delivered the HR data during the experimental training sessions.

### Statistical Analysis

All the data were presented as means ± standard deviations. The distribution of the data was assessed using the Shapiro-Wilk test. The Levene’s test was applied to check the homogeneity of variance. After dividing participants into two experimental groups, the initial test results were compared between the PD_90_ and the PD_180_ group using the *t*-test for independent variables. Analysis of covariance (ANCOVA) was performed to identify the between-group differences with controlling the covariate (pre-intervention measurements). Partial eta square (*_p_*η^2^) was calculated to determine the effect size (ES). ES values were defined as small (≥ 0.01), medium (≥ 0.06), or large (≥ 0.14) (Cohen, 1988). The level of significance was set at *p* ≤ 0.05. All analyses were performed using Statistica 13.0 software (TIBCO Software Inc., 2017 Palo Alto, CA, USA).

## Results

**Figure 1:**
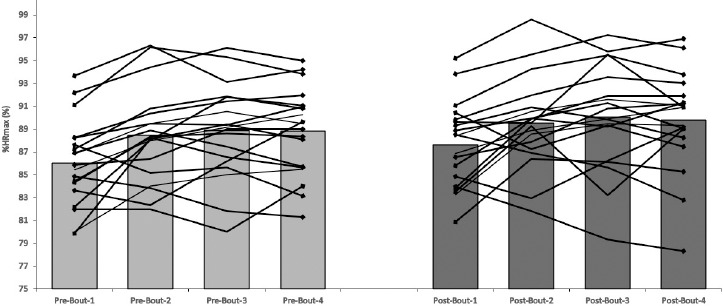
Individual and group heart rate responses during the first (PRE-) and the last (POST) intervention training sessions in the PD_90_ group.

**Figure 2:**
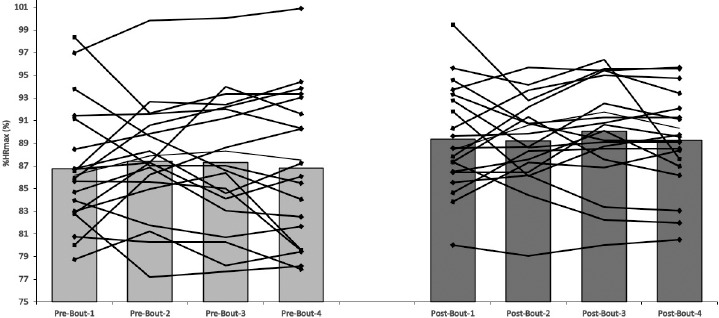
Individual and group heart rate responses during the first (PRE-) and the last (POST) intervention training sessions in PD_180_ group.

The average intensity of SSGs was between 86.4 and 89.1% HR_max_ for the PD_90_ group and between 85.6 and 89.2% HR_max_ for the PD_180_ group. No significant within-group differences were reported in HR responses during subsequent SSGs (Figures 1 and 2).

No significant differences between physical fitness variables were reported at baseline. ANCOVA revealed significant between-group differences after the intervention in the PWC_170_ (*p* = 0.018, ES = 0.15) and the 5-m sprint (*p* = 0.002, ES = 0.24). Other analyzed variables did not show any significant differences. All changes in fitness variables are presented in Table 2.

**Table 2:** Physical fitness variables of PD_90_ and PD_180_ groups before (PRE) and afer (POST) the eight-week intevention.

	PRE		t-test (*p*)	POST		ANCOVA *p* (η*_p_*^2^)
	PD_90_	PD_180_		PD_90_	PD_180_	
PWC_170_ (kGm•min^−1^)	18.9 ± 3.3	19.5 ± 3.3	0.60	18.1 ± 2.9	20.4 ± 3.2	0.018 (0.15)
VO_2max_ (ml•kg•min^−1^)	51.6 ± 6.7	51.6 ± 6.6	0.99	50.0 ± 7.0	52.9 ± 6.9	0.096 (0.07)
HR/LT (b•min^−1^)	171.6 ± 7.7	174.7 ± 12.7	0.37	169.1 ± 7.8	170.4 ± 11.6	0.72 (0.03)
V/LT (m•s^−1^)	3.5 ± 0.1	3.6 ± 0.2	0.28	3.7 ± 0.2	3.8 ± 0.2	0.23 (0.04)
Peak power (W∙kg^−1^)	11.0 ± 0.5	11.4 ± 0.7	0.11	11.7 ± 0.7	11.8 ± 0.7	0.54 (0.10)
5 m (s)	1.16 ± 0.07	1.11 ± 0.08	0.07	1.05 ± 0.07	1.08 ± 0.07	0.002 (0.24)
30 m (s)	4.48 ± 0.18	4.33 ± 0.18	0.05	4.35 ± 0.17	4.21 ± 0.19	0.98 (<0.001)

PWC: physical work capacity; VO_2max_: maximal oxygen uptake; HR/LT: Heart rate at the lactate threshold; V/LT: velocity at the lactate threshold; _p_η^2^: partial eta square

Results of the analysis of time-motion responses during SSGs played during the first and the last intervention training session are presented in Table 3. ANCOVA did not reveal any significant between-group differences after the intervention. Nevertheless, the medium effect was reported for TD (*p* = 0.08, ES = 0.08) and WLK (*p* = 0.11, ES = 0.07).

**Table 3:** Time-motion responses of small-sided games performed on different pitch dimensions during the first (PRE) and the last (POST) intervention training sessions.

	PRE		*t*-test (*p*)	POST		ANCOVA *p* (_*p*_η^2^)
	PD_90_	PD_180_		PD_90_	PD_180_	
TD (m)	2430.1 ± 176.9	2557.7 ± 244.7	0.07	2463.3 ± 171.2	2679.0 ± 309.5	0.08 (0.08)
WLK (m)	204.3 ± 46.2	152.3 ± 31.3	<0.001	197.4 ± 36.7	200.8 ± 48.2	0.11 (0.07)
JOG (m)	729.8 ± 49.8	712.3 ± 82.6	0.43	679.9 ± 43.4	664.7 ± 88.7	0.93 (<0.01)
LSR (m)	825.4 ± 183.7	877.4 ± 185.7	0.39	949.8 ± 165.0	1014.3 ± 266.9	0.72 (<0.01)
HSR (m)	662.8 ± 105.2	754.5 ± 200.9	0.08	623.4 ± 146.1	721.9 ± 168.1	0.31 (0.03)
SPR (m)	4.7 ± 5.3	61.2 ± 28.2	<0.001	12.8 ± 11.2	78.4 ± 23.5	-

TD: total distance; WLK: walking distance; JOG: jogging distance; LSR: low-speed running distance; HSR: high-speed running distance; SPR: sprinting distance

## Discussion

This study aimed to compare the effects of two different pitch dimensions on physical fitness responses and time-motion characteristics in young soccer players. To the authors’ knowledge, this is the first study to evaluate the effectiveness of in-season game-based HIIT performed on different pitch areas. The main finding of the present study is that SSGs played on larger areas (such as 180 m^2^ per player) were more beneficial in developing aerobic capacity in comparison with small playing areas (e.g., 90 m^2^ per player). Furthermore, SSGs performed on smaller pitches improved the ability to accelerate at short-distance more effectively than games performed on larger playing areas.

SSGs played on pitches with various dimensions induce different psychophysiological and physical responses in young soccer players (Aslan, 2013; Clemente et al., 2022; Halouani et al., 2017). Despite some limitations, HR monitoring is widely used to evaluate physiological response, especially during high-intensity exercises (Casamichana et al., 2013; Rabano-Muñoz et al., 2023). The present research showed that average HR responses to SSGs induced 85.0–90% of HR_max_. Previous studies demonstrated similar results with intensity ranging from 86 to 91% HR_max_ (Casamichana and Castellano, 2010; Castillo-Rodríguez et al., 2023). In addition, the current analysis emphasized that HR responses decreased from 174.7 bpm to 170.4 bpm, with a reduction of 2.46 % in the PD_180_ group following the eight-week soccer-specific HIIT intervention. Our results demonstrate that a once-a-week soccer-specific HIIT intervention (lasting eight weeks) is effective in maintaining or even improving aerobic fitness levels of young soccer players during the in-season period. In a comparable study (Casamichana and Castellano, 2010), the mean HR responses of young soccer players were significantly higher during SSGs played on a larger (175 m^2^) in comparison to a smaller pitch size (~75 m^2^) (88.5% and 86.0%, respectively). Furthermore, in a previous study conducted on amateur soccer players, higher HR responses were found during SSGs played on a large pitch size (126 m^2^) compared to a medium (~88 m^2^) and a small (56 m^2^) pitch size (Rampinini et al., 2007). However, in contrast to the abovementioned findings, some previous studies demonstrated non-significant differences between SSGs played on large and small pitch sizes (Aslan, 2013; Randers et al., 2010). These contrary results could be caused by different methodological approaches related to HIIT. It has been previously recognized that such factors as the number of players or coach encouragement affect the intensity of the game (Rampinini et al., 2007). From a practical point of view, these findings can help coaches and sports scientists plan SSGs during the in-season period.

Numerous studies have recently shown that the manipulation of pitch sizes differentiates between time-motion characteristics in youth soccer players (Castillo et al., 2021; Castillo-Rodríguez et al., 2023; Clemente et al., 2022). TD values obtained in our research were comparable to those of Castillo et al. (2021) who registered in similar SSGs format TD ranging from 2191 to 2655 m for smaller and larger pitch sizes, respectively. Moreover, the results of the current study showed a significant group effect in TD (ES = 0.14) and distances covered in WLK (ES = 0.12), HSR (ES = 0.10), and SPR (ES = 0.78). Despite WLK, longer distance in these intensities was covered by players from the PD_180_ group. These observations are in line with several previous reports (Castellano et al., 2015; Castillo-Rodríguez et al., 2023; Olthof et al., 2018). Larger pitch dimensions enable players to reach higher velocities during the game. Hence, distance covered in HSR and SPR is greater when SSGs are played on bigger fields (Riboli et al., 2020). These differences in acute time-motion responses could result in some chronic adaptations. On the other hand, SSGs on small or medium fields could lead to an increased number of short-distance accelerations (Guard et al., 2021). This relationship may explain significantly better sprint time at the distance of 5 m reported in the PD_90_ group after the eight-week intervention.

The nature of soccer is characterized by a combination of low-, high- and moderate-intensity actions during the game. Therefore, V/LT responses to high-intensity game-based training are thought to be as important as the change in VO_2max_ responses (Edwards et al., 2003; Ziogas et al., 2011). The present study results showed more beneficial changes in PWC_170_ in the P_180_ group. These improvements suggest that SSGs performed on a larger pitch could be more beneficial in developing aerobic fitness of young soccer players. Furthermore, V/LT values increased by 0.2 m•s^−1^ after the intervention in both groups. These different responses could be explained by the fact that V/LT is recognized to be a more sensitive marker of aerobic fitness in comparison with VO_2max_ (Coyle, 1995; Grant et al., 1997). Moreover, one HIIT session a week could be an insufficient stimulus to significantly improve maximal oxygen uptake in young soccer players. Similarly, the results of ANCOVA did not reveal significant interactions in such variables as HR/LT, V/LT, peak power, 30-m sprint distance and time-motion variables. However, medium ES was observed in peak power, TD and WLK (0.10, 0.08 and 0.07, respectively). Only one training session a week including SSGs was probably not sufficient to reach the significance level of all analyzed physical fitness variables.

Current research is the first attempt to analyze the effect of an in-season SSG intervention in young soccer players. Despite this novelty, some limitations are worth mentioning. The assessment of aerobic capacity was performed using the submaximal test on the cycle ergometer. Due to the specificity of movement, running tests are considered to be more advisable for soccer players. However, from the practical perspective, coaches often are not willing to apply numerous all-out efforts to their players during the in-season period. Furthermore, the lack of potentially important psychophysiological assessment (e.g., the RPE or the physical activity enjoyment scale) could be considered a limitation as well. Finally, the number of intervention training sessions seems to be questionable. Nevertheless, during the league competition, when teams play official games every 6–7 days, it is difficult to implement a greater number of game-based HIIT training sessions.

The main findings of the current study indicate that SSGs performed once a week could effectively maintain or even improve the level of aerobic fitness and 5-m sprint time in young soccer players during the in-season period. Greater playing areas could be more beneficial in developing aerobic fitness, while game-based training on smaller pitch sizes improves the ability to accelerate at short distances. Therefore, coaches and fitness trainers should be aware that in-season game-based HIIT on different field sizes could result in specific changes in physical and time-motion response in young soccer players.
